# Computational strategies for cross-species knowledge transfer and translational biomedicine

**Published:** 2024-08-16

**Authors:** Hao Yuan, Christopher A. Mancuso, Kayla Johnson, Ingo Braasch, Arjun Krishnan

**Affiliations:** Genetics and Genome Science Program; Ecology, Evolution, and Behavior Program, Michigan State University; Department of Biostatistics & Informatics, University of Colorado Anschutz Medical Campus; Department of Biomedical Informatics, University of Colorado Anschutz Medical Campus; Department of Integrative Biology; Genetics and Genome Science Program; Ecology, Evolution, and Behavior Program, Michigan State University; Department of Biomedical Informatics, University of Colorado Anschutz Medical Campus

## Abstract

Research organisms provide invaluable insights into human biology and diseases, serving as essential tools for functional experiments, disease modeling, and drug testing. However, evolutionary divergence between humans and research organisms hinders effective knowledge transfer across species. Here, we review state-of-the-art methods for computationally transferring knowledge across species, primarily focusing on methods that utilize transcriptome data and/or molecular networks. We introduce the term “agnology” to describe the functional equivalence of molecular components regardless of evolutionary origin, as this concept is becoming pervasive in integrative data-driven models where the role of evolutionary origin can become unclear. Our review addresses four key areas of information and knowledge transfer across species: (1) transferring disease and gene annotation knowledge, (2) identifying agnologous molecular components, (3) inferring equivalent perturbed genes or gene sets, and (4) identifying agnologous cell types. We conclude with an outlook on future directions and several key challenges that remain in cross-species knowledge transfer.

## Introduction

The use of research organisms, also known as model species or model systems, is essential to biomedical research [1]. Leveraging their resemblance in anatomy, physiology, behavior and genetics to corresponding human conditions, research organisms help scientists investigate a wide range of biomedical phenomena and therapeutic treatments before they are applied to humans. For instance, the zebrafish (*Danio rerio*) is a well-established vertebrate research organism that has external and fast development in large numbers, transparent embryos, and allows for easy genetic manipulation and drug administration through compound exposure [2].

There are two main reasons that research organisms are critical to studying human phenotypes, processes, and diseases. First, it is often unethical to study biomedical processes or apply novel therapeutic treatments directly in humans. Although there are many unanswered questions on the ethics of using research organisms [3,4,5], the knowledge derived from these organisms has helped save countless human lives [2,6]. Secondly, genetic studies in humans typically have very high variability due to confounding effects such as population genetics and environmental factors [7,8,9,10], and these confounders can be controlled much more in laboratory research with research organisms.

However, using research organism data to explore human biology presents its own challenges. Evolutionary divergence often gives rise to similar yet different underlying biological processes between human and research organisms [11], thereby impeding the transfer of knowledge across species. Even orthologs (i.e. homologous genes across species), which have taken a privileged role in transferring functional annotations across species, may have significant functional changes across species [12,13,14,15,16,17]. Additionally, the complex genetics underlying phenotypes/processes shared across species may differ. This is because interactions among genes responsible for a phenotype could be rewired during the evolutionary process, potentially encompassing some species-specific genes [18]. Consequently, the effective and precise transfer of knowledge considering species-specific genes remains challenging. Finally, since no single research organism can fully recapitulate the entirety of a complex biological condition in humans [1], how do we predict which research organisms capture the different facets of the human biology of interest?

To address these issues, researchers have begun developing computational models that can robustly transfer knowledge between a variety of research organisms and humans to identify functionally similar genes or groups of genes across species regardless of their evolutionary origin. For functionally equivalent genes that may or may not be orthologous, we here coin the term “agnolog”. We define agnologs to be biological entities, processes, or responses — e.g., genes, gene sets, or even biological systems — that are “functionally equivalent” across species regardless of evolutionary origin. “Agno-” conveys the sense of being a data-driven observation that is noncommittal about the evolutionary origin (may they be homologous or convergently evolved) and compatible with any biological explanation. Recent discoveries of agnologs were evidenced by large-scale competitions such as sbvIMPROVER Species Translation Challenge [19] and the Critical Assessment of Protein Function Annotation Challenge (CAFA) [20,21,22,23].

**Figure 1:**
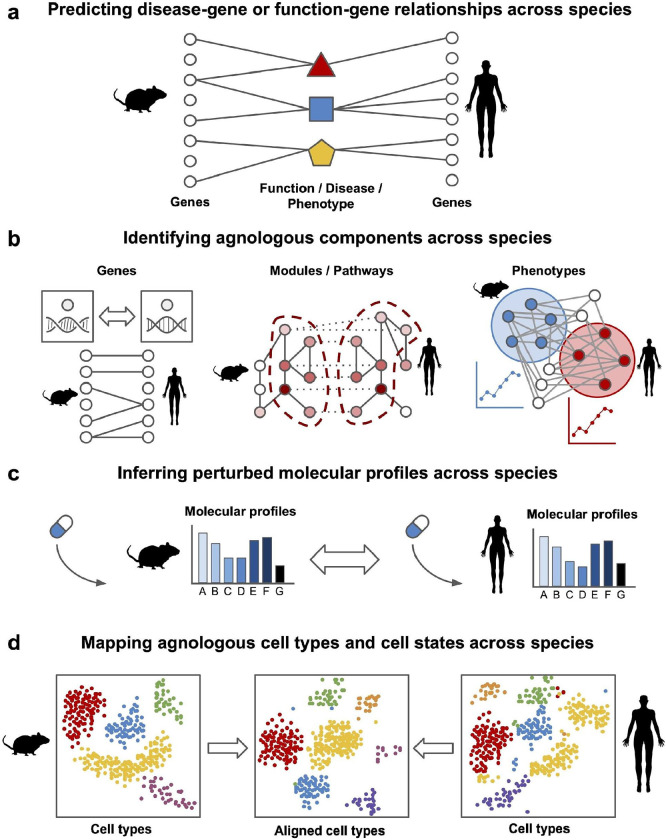
Schematic diagram of each section of this review article. **a.** How to predict disease-gene or function-gene relationships across species? **b.** How to identify agnologous molecular components across species? **c.** How to infer perturbed molecular profiles across species? **d.** How to map agnologous cell types and cell states across species?

In this review, we present a suite of cross-species knowledge transfer approaches with a significantly broader scope than previous reviews [24,25,26,27]. We comprehensively lay out the landscape of recent and state-of-the-art data-driven strategies, including those that leverage AI and machine learning, to answer four classes of important questions that frequently arise when using research organisms to study biomedical questions and translating findings to humans (Figure 1): **(1)** How to predict disease-gene or function-gene relationships across species? **(2)** How to identify agnologous molecular components across species? **(3)** How to infer perturbed molecular profiles across species? **(4)** How to map agnologous cell types and cell states across species?

Instead of discussing methods through an algorithmic lens, we are taking a question-first perspective to provide inspiration and background both for computational researchers interested in developing new methods in this area and for experimental/wet-lab researchers interested in finding and using the best methods in this area. Although we have separated the methods into four sections, many of the approaches described across these sections share similarities in algorithmic design, data types, and output formats. Detailed information about the methods discussed can be found in ***Supplementary File 1***, presented for researchers to easily find the methods and tools relevant to their application of interest. In addition, we have curated the benchmark datasets used in the discussed methods, summarized in ***Supplementary File 2*** and ***Supplementary Note 1***. These resources will enable researchers to test and improve upon existing computational methods in the field.

## How to predict disease-gene or function-gene relationships across species

**Figure 2:**
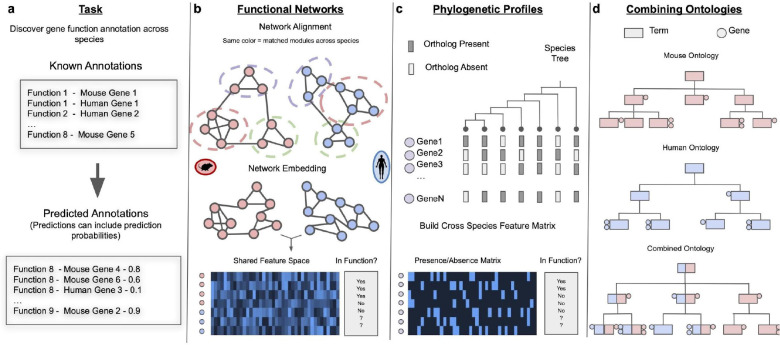
How to predict disease-gene or function-gene relationships across species. **(a)** The objective is to predict gene-related functions or diseases across species by leveraging known annotations. **(b)** Network-based methods can annotate diseases or functions for genes across species by aligning networks or by embedding networks into a shared, low-dimensional feature space where a supervised learning model is then trained to propagate annotations. **(c)** Phylogenetic profile-based methods identify co-presence or co-absence of genes throughout evolutionary history, implying closely related functions among these genes. This relationship is then used to propagate annotations across species. **(d)** Disease and function annotations can be transferred by combining ontologies across species. Genes with similar functions or disease associations across species can then be identified through the ontology structure.

Comprehensive knowledge of the roles genes play in molecular functions, phenotypes, and diseases is fundamental to our understanding of the molecular underpinnings of biological, physiological, and pathological processes. However, the roles of less than half of all genes in the human genome have been experimentally characterized even in a single biological context. Knowledge of gene-function, gene-phenotype, and gene-disease relationships is significantly richer, though far from complete, in research organisms due to the availability of a variety of experimental techniques such as gene editing, knock-in, and knockout experiments [28,29]. How do we leverage this knowledge available across species to close massive annotation gaps, especially in light of the potential functional divergence of homologous genes and the presence of species-specific genes? Moreover, how do we use existing knowledge in human and traditional research organisms (e.g., mouse, frog, zebrafish, fly, worm) to fill annotation gaps in non-traditional research organisms (e.g., dog [30], python [31], gar [32], planaria [33]) that lack sufficient data. These needs have spurred the development of a number of computational methods for predicting disease-gene or function-gene relationships across multiple species (Figure 2).

### Utilizing molecular networks to predict functional gene annotations across species

Molecular interaction networks composed of experimentally verified or computationally predicted physical and/or functional relationships between pairs of genes (or their products) have become indispensable tools for predicting novel gene annotations based on the principle of “guilt-by-association”, which states that genes close to each other in the underlying molecular network will be involved in similar biological processes [34]. Interaction neighborhoods within these networks capture the “functional context” of genes, which is highly complementary to sequence homology information. Further, these networks typically contain and therefore enable making inferences about nearly all genes in the genome, including un(-der)studied and species-specific genes. Therefore, a number of network-based computational approaches have been developed to transfer and predict function, phenotype, and disease annotations across species, especially using machine learning (ML) approaches [35,36,37,38,39,40].

For instance, the Functional Knowledge Transfer (FKT) method [36] first finds “functionally-similar homologous gene pairs” that are in the same gene family and in similar network neighborhoods [41], and uses these pairs to propagate functional annotation across species. FKT significantly enhanced the prediction of gene-pathway associations, especially for biological processes lacking extensive experimental data in the target organism, enabling the transfer of functional insights from well-studied organisms to less-explored ones. For example, by transferring knowledge from other species, FKT successfully annotated the genes related to the regulation of exit from mitosis (GO:0007096) in zebrafish, which had no experimental annotation at the time of the study. One of the top genes predicted by FKT, *cdh2*, has then been experimentally confirmed related to cell cycle progression in zebrafish retina cells [42].

NetQuilt [37] addressed the challenges of transferring functional gene annotations across species by integrating multiple networks across species using IsoRank [43], which aligns networks considering both sequence similarity and network similarity. To predict annotations across species, NetQuilt utilized a deep learning (DL) model trained on node representations of annotated genes from one or multiple species to predict a fixed set of annotations for genes in the rest of the species.

GenePlexusZoo [38] simultaneously integrates networks of human and five research organisms (mouse, zebrafish, fly, worm, yeast) to generate a “functional representation” of genes from all these species that can be used for any pathway, phenotype, or disease prediction task. By training ML models on this joint multi-species network representation, GenePlexusZoo improves the performance of predicting gene annotations within a single species as well as facilitates knowledge-transfer across species.

### Predicting candidate disease genes using networks

While, conceptually, the aforementioned methods can be used to also predict disease-gene relationships, other network-based methods have been developed exclusively for predicting disease associations based on cross-species information.

Katz measure [44], a well-established technique in social network link prediction [45], has been successfully applied to discover connections between diseases and genes across species [46]. The authors constructed a heterogeneous network that included gene-gene, gene-disease, and disease-disease links within humans and then added links between genes in humans and other species based on sequence homology. Then, novel disease gene candidates were identified as those in close proximity to disease nodes in this network (based on overall path lengths), estimated using the Katz measure. Further, “Katz features” that represent the number of paths of a certain length between a gene and a disease node, when incorporated into a ML framework, were shown to significantly enhance the performance of predicting gene-disease relationships [46].

DiseaseQUEST [47] predicts disease-gene candidates in research organisms by combining human genome-wide association studies (GWAS) [48] with gene networks that reflect pathways underlying tissue-specific physiology [49] and disease [50,51]. Specifically, in a target species, using homologs of GWAS disease genes as positive examples and gene interactions in an appropriate tissue network as features, diseaseQUEST trains an ML model to prioritize novel disease-gene candidates. The authors demonstrated this approach by prioritizing and experimentally validating genes related to Parkinson’s disease in the nematode *Caenorhabditis elegans* [47].

In addition, numerous methods have been developed for predicting disease-gene associations in single species, typically in humans. These methods can be adapted to predict disease-gene associations across species [39,52,53,54,55].

### Discovering disease-related genes using phylogenetic profiles

Genes with similar functions tend to appear and disappear jointly during the evolutionary process. Most human disease genes have ancient origins [56,57,58,59], with many traceable to eukaryotic ancestors, some dating back to the evolution of multicellularity [56]. By identifying genes that co-evolve with known disease-related genes, we could propagate the disease annotations from the known genes to the co-evolved genes, thereby helping predict functions for genes that are not well characterized [60,61].

Maxwell and colleagues [62] used evolutionary profiles to examine the evolutionary distribution of human disease genes from the Online Mendelian Inheritance in Man (OMIM) database [63]. They revealed heterogeneity underlying the evolutionary origins of different classes of human disease genes. For example, genes related to inflammatory and immune diseases are of vertebrate origin, while genes associated with cardiovascular and hematological diseases originate from early metazoans.

Recent methods [64,65] enabled systematic screens of genome-wide co-occurring functional modules, leading to functional predictions for numerous previously uncharacterized genes. For example, using phylogenetic profiling, Dey et al. [65] identified understudied candidate genes linked to the actin-nucleating WASH complex and ciliary and centrosomal defects by scanning co-occurring gene modules across 177 species on the eukaryotic species tree. They further evaluated candidate functions of genes by identifying gene product co-localization and gene knockdown.

### Bridging disease-gene association through phenotypic similarity

Extensive gene-phenotype associations in research organisms have been discovered through hypothesis-driven experiments and large-scale genetics screens [66,67,68]. These links can lead to the identification of genes in a research organism whose perturbation results in phenotypes similar to those observed in patients with a particular disease, thereby pointing to a viable model for the disease under observation.

However, comparing phenotypes across species is challenging due to evolutionary divergence and non-standard descriptions of phenotypes in different species. The continuous efforts in phenotypic ontology curation and the development of cross-species phenotype ontologies such as uPheno [69] and PhenomeNET [70] have now significantly mitigated this challenge, enabling researchers to use ontology-based semantic similarity measurements between phenotypes to explore suitable research organisms and identify new disease-gene and function-gene relationships. For instance, Exomiser [71,72] leveraged disease-to-gene relationships discovered through semantic similarity measures among integrated ontologies to assist in disease diagnosis.

Recent studies improve on semantic similarity by creating a latent embedding space based on the phenotype ontology combined with known disease-phenotype and disease-/phenotype-gene associations[73,74]. “Node embeddings” created this way contain a low-dimensional numerical representation of each entity, such as gene, phenotype, and disease that captures that entity’s relationships. Such embeddings naturally lend themselves as inputs into ML algorithms. In this study [73], the authors trained a supervised neural network model to predict gene-disease associations based on the node embeddings of genes and diseases, which performed better than an unsupervised approach based on the similarities between gene and disease embeddings.

## How to identify agnologous molecular components across species

**Figure 3:**
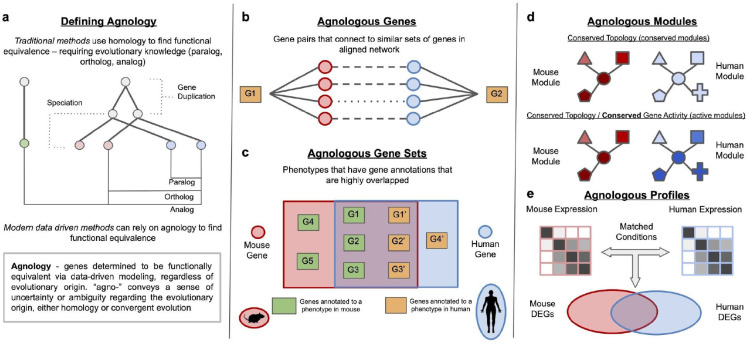
How to identify agnologous molecular components across species. **(a)** Definition of agnology. **(b)** Agnologous gene pairs can be identified through genes that connect to similar sets of genes in aligned cross-species gene networks. **(c)** When prior knowledge about gene sets is known, agnologous gene sets are discovered through large overlaps between gene sets across species. **(d)** When prior knowledge is not available, agnologous gene sets can be discovered by finding sub-networks with similar topology and expression patterns across species. **(e)** Resemblance at the organismal level, i.e., agnologous profiles, can be examined by comparing genomic profiles across species.

Complementary to inferring genes in each species that are associated with a particular function or disease, another critical task is to infer molecular components that are equivalent counterparts of each other across species. Termed “agnologs” above, these molecular counterparts reveal novel biology when they are inferred in a data-driven manner, in the context of a specific condition or perturbation, and regardless of their evolutionary origin (e.g., homology). A number of computational approaches have been developed to identify such agnologous components at the gene, pathway (gene set), and genomic level across species (Figure 3).

### Identifying agnologous gene pairs across species

An essential ingredient of finding agnologous genes is to go beyond solely using sequence similarity and homology because, in many organisms, proteins performing similar functions (e.g., playing similar roles in the same biological pathway) may not be the most similar in sequence [75]. Gene Analogue Finder implemented this notion by measuring functional similarity between a pair of genes based on the overlap between gene ontology (GO) terms associated with them [76]. Han et al. [15] used a comparable approach to calculate functional similarity between homologous human-mouse gene pairs based on the average pairwise similarity between human and mouse phenotype ontology terms annotated to those genes. This study identified several cases of functional divergence of orthologs that could be traced to changes in noncoding regulatory sequences of gene pairs with high protein sequence similarity. Nevertheless, the performance of such methods depends heavily on the completeness of gene annotations to terms in function and phenotype ontologies, with term overlap estimates becoming less meaningful for particular genes or entire species with sparse (i.e., highly incomplete) annotations [15].

Genome-scale gene interaction networks help overcome this limitation by providing a powerful alternative way to capture the “functional context” of each gene in terms of its local network neighborhood. Thus, two genes across species interacting with similar sets of genes in their respective molecular network neighborhoods are likely agnologs. Chikina et al. [41] realized this idea by first grouping the network neighbors of individual genes into meta-genes that correspond to Treefam families [77] and measuring the hypergeometric overlap between sets of meta-genes that are neighbors of a pair of genes across species in the respective species network. Intersecting meta-genes identified through this approach revealed specific biological processes underlying network similarities.

Other recent network-based approaches for finding agnologous genes take advantage of the idea of node embedding. Methods such as MUNK [78], MUNDO [79], and ETNA [80] create a joint network-based representation of genes by embedding the molecular networks in a pair of species individually and then align the two embeddings based on sequence orthologs. MUNK produces different joint embeddings based on the “source” species and the “target” species, where the joint embedding is as large as the number of genes in the smaller network. ETNA uses cross-training to align the two embeddings into a bidirectional compressed (low-dimensional) space. MUNK, MUNDO and ETNA use the similarity of embedding vectors of genes to estimate their functional similarity or to inform function or disease gene prediction.

### Discovering agnologous gene sets across species

Going beyond gene pairs, it is also of interest to find functionally conserved gene sets across species because sets of genes could represent concepts like pathways and molecular mechanisms underlying conserved phenotypes. McGary et al. [81] introduced the concept of orthologous phenotypes, i.e. “phenologs”, defined as phenotypes that are associated with orthologs. Phenologs could include phenotype counterparts that are observably very different from each other while being influenced by conserved molecular functions (e.g., significantly overlapping sets of orthologous genes). Examples include a yeast model for angiogenesis defects, a worm model for breast cancer, mouse models of autism, and a plant model for human neural crest defects [81]. Phenologs serve as a valuable tool to quantitatively identify non-obvious research organism phenotypes for studying human diseases, along with disease gene candidates such as genes annotated to non-obvious phenotypes and which are not orthologous to any known disease genes.

### Discovering agnologous gene modules across species

Phenolog approaches rely on prior knowledge of functional and phenotypic annotations of genes, which is often highly incomplete. Network-based methods have proven valuable in overcoming this limitation by helping to find conserved gene sets, usually called gene modules, while filling in knowledge gaps. This is because, in addition to capturing relationships between pairs of genes, networks also capture higher-level organization between groups of genes in the form of tight sub-networks. Consequently, similar sub-networks across species correspond to homologous functional modules or pathways. CoCoCoNet [82] takes such a network-based approach to test whether a given gene set in one species is involved in similar functions as homologous gene sets in another species. If a subset of genes accurately predicts the remaining genes in the gene set using neighbor voting in both species, that is taken as an indication that the gene set corresponds to a conserved functional module.

Molecular networks can further refine comparisons of differential gene expression across species to help identify functionally conserved “active modules” that comprise tightly connected sub-networks of conserved genes that respond similarly to a given condition (e.g., disease, perturbation). However, finding such modules is difficult because active modules in different species are often not conserved across species, while conserved modules are not necessarily active [83]. So, algorithms for finding conserved and active modules need to consider activity plus conservation at the same time.

The neXus algorithm [84] was developed to meet this need. neXus identifies modules using a greedy seed-and-extend algorithm. It begins with a pair of orthologous nodes as seeds and iteratively extends both sub-networks by incorporating neighboring orthologous gene pairs. ModuleBlast [85] works similarly to neXus with the added capability of distinguishing the resulting modules based on the direction of the expression change. This separation allows ModuleBlast to evaluate whether conserved active modules display expression patterns in the same or opposite way. Both neXus and ModuleBlast limit the identification of modules to fully conserved genes. xHeinz[83] relaxes this constraint by allowing users to define the proportion of conserved nodes, offering a more flexible approach that leads to including functionally conserved but non-homologous (even species-specific) genes in the final modules. xHeinz was further applied to understand the regulatory mechanism of interleukin-17-producing helper T (Th17) cell differentiation in humans and mice. The study revealed that key regulators of Th17 cells are conserved across species [83].

### Measuring agnologous profiles between human and research organisms

Beyond genes and gene modules, evaluating the ability of a research organism to mimic specific human biological processes is crucial for functional studies and experimental design. However, such evaluations are subjective, with researchers often using vague terms such as “poorly” or “greatly” to indicate resemblances. Therefore, some recent methods have leveraged functional genomics data to draw quantitative conclusions about the biological resemblance between human and research organisms.

Congruence Analysis for Model Organisms (CAMO) [86] quantitatively measures the congruence between human and research organisms by comparing the distribution of differentially expressed gene (DEG) profiles under matching conditions. To improve the accuracy of identifying DEGs, CAMO infers differential posterior probabilities of genes based on p-values from conventional pipelines. The concordance level of differential gene expression across species was summarized as concordance (c-scores) and discordance scores (d-scores), which served as quantitative measures of congruence across species. CAMO was applied to reconcile studies [87,88] that reached contradicting conclusions about whether mice are a suitable model for studying human inflammatory diseases. By reanalyzing and comparing inflammatory expression data within and across species, the authors concluded that burn- and infection-induced inflammation in mice resembles human inflammation [86].

Comparing phenotypes provides another avenue to discover biological resemblance in research organisms. Cross-species phenotypic ontologies can link phenotypes related to human diseases to research organism phenotypes. Leveraging such connections, the Monarch Initiative [72] provides tools, such as the Phenotype Explorer, to prioritize research organisms based on phenotypic similarities, aiding in the discovery of relevant research organisms and phenotypes.

## How to infer perturbed transcriptomes across species

**Figure 4:**
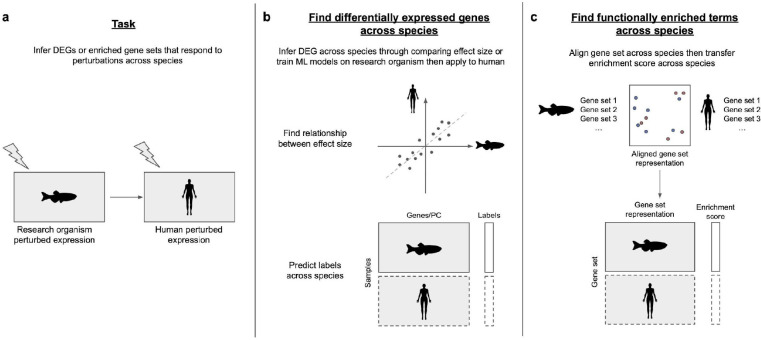
How to infer perturbed transcriptomes across species. **(a)** The objective is to infer potential perturbed genes or gene sets in humans based on experimental perturbation results in research organisms. **(b)** To infer DEGs across species, FIT [89] uses linear models to describe relationships between the effect sizes of DEGs in humans and research organisms with matching conditions. Other approaches develop ML models based on research organisms to predict perturbed expression in humans. **(c)** To infer enriched gene sets across species, XGSEA [90] aligns gene set representations across species, train models in research organisms, and then infer enrichment scores of gene sets across species.

Bulk and single-cell transcriptome profiling have emerged as preeminent technologies for nearly any organism to capture genome-wide molecular responses to a variety of developmental and physiological states as well as treatments, perturbations, and other conditions. Transcriptome profiling in research organisms has especially been valuable in studying perturbations that may be impractical or ethically infeasible in humans. Further, comparing transcriptomic profiles across species sheds light on conserved and distinct cellular states and gene responses, and makes way for context-specific knowledge transfer. However, directly comparing expression changes across species based on gene homology is challenging due to evolutionary divergence in gene expression programs. This section describes several methods that have been developed to utilize computational techniques to enable accurate comparisons of transcriptomic profiles across species (Figure 4).

### Determining differentially expressed genes across species

Identifying differentially expressed genes (DEGs) from transcriptome profiles has led to insights into the impact of numerous diseases, perturbations, or experimental conditions. To systematically capture the relationship of DEGs across species, a class of methods train ML models using carefully constructed cross-species dataset pairs (CSDPs) with matching conditions, offering curated examples of how expression changes correspond to phenotypic outcomes across species, which can be further used for model training [89,91].

Found In Translation (FIT) [89] is a linear regression model that is used to understand relationships of DEGs between human and mice for each orthologous gene pair. The authors built models based on 170 mouse-human CSDPs across 28 conditions, which were pairs of mouse and human experiments associated with the same disease or condition. The model was then used to predict DEGs in humans under conditions analogous to those captured in novel mouse data. FIT identified more human DEGs compared to simple transfers of DEGs from research organisms to human based on orthology. For instance, using protein immunostaining, the authors confirmed their prediction that the gene *ILF3* is upregulated in the colon of patients with inflammatory bowel diseases (IBD) even though the gene was not differentially expressed in either human or mouse data [89].

The FIT approach is likely to be limited to diseases or drug perturbations with sufficient training data from both human and research organisms. In contrast, Brubaker et al. [91] proposed a semi-supervised method to predict DEGs across species that only requires phenotype information in research organisms. Initial supervised models were built to predict mouse phenotypes using mouse expression data, and then these models were iteratively augmented with high-confidence human samples to predict the phenotypes of the remaining human expression data. Finally, differential gene expression analyses were performed on human samples with distinct predicted phenotypes. Transcomp-R [92] was proposed as an alternative approach to predict DEGs across species when phenotype labels are available in only one species. Its prominent application involved finding mouse genes that are predictive of infliximab responses in chronic IBD patients when corresponding phenotype labels are not available in mice. After representing murine expression data in a low-dimensional space using principal component analysis (PCA) and projecting human expression data into the mouse PC space, Transcomp-R identified murine PCs (and corresponding genes) most associated with infliximab response by regressing the mouse PCs of the projected human data against the human phenotype labels. This approach identified the activated integrin pathway signaling in IBD patients with infliximab resistance. Single-cell sequencing on patient biopsies revealed over-expression of one of the top genes, *ITGA1*, in immune cells, which probably mediates infliximab resistance. The function of *ITGA1* in immune cells was further experimentally validated by treating anti-ITGA1 on patients’ peripheral blood mononuclear cell samples [92].

Finally, other recent work [93,94] includes treating the biological condition such as a tissue or a disease as a “style” and then utilizing style transfer techniques to predict transcriptomic changes across conditions within the same species. Though promising, it is important to note that genetic differences across species are significantly larger and more complex than the condition changes observed within a single species. As a result, applying style transfer techniques directly to cross-species comparisons may face additional challenges and limitations that need to be carefully considered and addressed.

### Determining functionally enriched terms across species

Gene set analysis (GSA) has been widely used to identify predefined gene sets (e.g. Gene Ontology biological processes) that are significantly enriched in a gene list of interest [95]. GSA provides a powerful approach to gaining insights into enriched functional terms associated with diseases, phenotypes, or perturbations. However, the discrepancy of genes across species makes the definition of functionally equivalent gene set across species difficult, so directly transferring enriched terms across species is impractical.

XGSEA [90] tackles the challenge of transferring enriched terms across species by using affine mapping, which projects genes from humans and research organisms into the same space. In this joint space, gene sets sharing more homologous genes will be closer to each other in the transformed space. With the transformed gene set representations, logistic regression models were built to characterize the relationship between the representation and enrichment metrics from the research organism, including p-values and enrichment scores of gene sets. Finally, the trained models are used to predict enrichment metrics for gene sets from humans. XGSEA successfully estimates enrichment metrics in human gene sets based on metrics of homologous gene sets in research organisms. For instance, XGSEA was able to train models based on zebrafish data and predict enriched pathways associated with melanomas in human patients [90].

## How to map agnologous cell types and cell states across species

The advent of single-cell and single-nucleus sequencing techniques has opened up new avenues in biological research. This emerging frontier focuses on identifying and comparing cell types and states across different species to better understand the cellular diversity and species-specific cellular innovation arose from cell type evolution [96,97]. By transferring insights from extensively studied organisms to less-explored ones, these cross-species comparisons offer a powerful insight for cell type identification and discovery, thereby advancing our understanding of cellular biology across different species.

**Figure 5:**
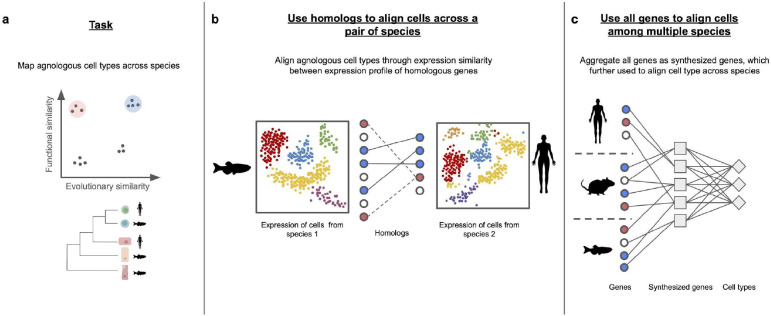
How to map agnologous cell types and cell states across species. **(a)** The objective is to align analogous cells (represented by points shaded in red and blue patches) across species, given pairs of single-cell datasets or even multiple single-cell datasets from different species. **(b)** The majority of methods align cells with similar expression patterns in homologs across a pair of species. **(c)** SATURN [98] stands out as the only method capable of aligning single-cell datasets from multiple species simultaneously. It first aggregates genes into synthesized genes using PLM [99]. These synthesized genes are then utilized to align cells across multiple species.

Cell mapping problems may be simply conceptualized as mapping transcriptionally similar cells across species (i.e., based on homologous genes having similar expression levels). However, cells of a particular type within a species are more alike than those of corresponding cell types across different species. [100,101]. Therefore, effectively mapping cells across species requires correcting for species-specific expression differences so that cells of homologous types are mixed in irrespective of species origin, while cells of different types remain distinct from each other. Additionally, methods must account for experiment-induced batch effects. Some tools have been developed to reconcile heterogeneous single-cell RNA-seq (scRNA-seq) data from multiple species [100,102,103,104,105]. These methods typically seek to project cells from multiple species into a unified space, facilitating cross-species comparison. As a result, the cell mapping problem can be viewed as a domain adaptation problem, i.e., aligning cells from different species in a unified space (Figure 5).

### Aligning cells across a pair of species based on homologs

Shafer [97] summarized various methods for cross-species scRNA-seq integration. Originally designed to mitigate batch effects introduced by experimental variations, these methods may not sufficiently correct for species differences, which are more pronounced than batch effects [101]. When comparing human cells to those of anciently polyploid species like the teleost fish, the abundance of gene duplicates results in fewer one-to-one gene matches, leading to a reduced signal for alignment. Moreover, orthology does not necessarily imply similar functions across species, and paralogs might exhibit more functional similarity than orthologs. For instance, when an ortholog acquires a loss-of-function mutation, its function might be compensated by the upregulation of a paralog, resulting in the paralog having a more similar function to the ortholog in the other species [106,107]. Therefore, incorporating one-to-many or many-to-many homology into the analyses could improve cross-species alignment performance by accounting for functional similarity between paralogs.

In a benchmark study conducted by Song et al. [108], SAMap [109] was identified as the only method that is capable of mapping divergent cell types. To account for the effect that expression patterns may be divergent in homologous cell types, SAMap uses the full gene homology information and incorporates neighboring cells within species into the calculation of similarity between cells across species. Consequently, cells with differing expression profiles remain closely associated if they are among the nearest cross-species neighbors to each other. Based on this analysis, SAMap identified general alignment between gene expression patterns and developmental relationships during embryogenesis in frogs and zebrafish. Interestingly, they also detected a group of secretory cell types that have similar expression patterns while having different developmental origins including arising from different germ layers [109].

### Aligning cells among multiple species using all genes

Instead of using precalculated gene homology, a new framework SATURN [98] integrates cell atlases from divergent species by harnessing the power of protein language models (PLMs) to relate genes across species to each other and project all cells from multiple species into shared cell embedding space. PLMs can learn informative representations of protein sequences that can implicitly reflect similarity of function, protein structure, molecular characteristics [110], as well as evolutionary relationships [104] between proteins. Using a neural network, SATURN first projects scRNA-seq datasets to a joint space composed of “macrogenes” representing groups of genes across species that have similar protein embeddings inferred from the PLM. The neural network weights is used to define the importance of a gene to a macrogene. For each macrogene, SATURN learns the cross-species cell embedding as a non-linear combination of macrogenes, guided by an unsupervised objective function that simultaneously maximizes the distance between distinct cells within the same species and minimizes the distance between similar cells across species. This approach enables SATURN to integrate data from multiple species (and not just perform pairwise comparisons), including divergent species where gene homology relationships are hard to unravel. The output of SATURN also enables easy cross-species comparison, such as finding differentially-expressed macrogenes across species, which can then be traced back to actual genes based on the weight of connections to the macrogenes in the neural network. SATURN’s application to a multi-species dataset of frog and zebrafish embryogenesis shows success in aligning evolutionary-related cell types and revealing differentially expressed genes in macrophage/myeloid progenitors and ionocytes across these two remotely-related species [98].

## Future perspective

The explosion of large-scale human biological data and accompanying analysis methods, while seen as a prospect of a golden age for human genetics, has also raised the question of whether it will diminish the significance of research organisms [7,111]. From the perspective of this review, the answer is certainly “No”. In fact, the ocean of new data will lead to an increasing number of candidate disease-related genes, dysregulated pathways, drugs, etc., that will require validation and functional characterization, which can be carried out in completely in vivo settings only in research organisms [7]. Therefore, the goal of increasingly better computational strategies to effectively gain biological and biomedical insights from research organisms will continue to remain significant. The landscape of current methods, summarized in this review, demonstrates great promise towards this goal. Instead of solely relying on sequence similarity and gene homology, these approaches utilize sophisticated inferential techniques and diverse datasets, paving a promising path for effectively transferring knowledge between human and research organisms. The next frontier in unlocking the full potential of research organisms and the ever-growing datasets involves addressing some key challenges that still remain.

### Capturing the specific facets of complex diseases

Complex human diseases exhibit staggering genetic and phenotypic heterogeneity. Despite this complexity, much of the current focus is on identifying a single research organism that can fully recapitulate an entire disease. Consequently, a major need in the field is to develop methods that can identify optimal phenotypes, conditions, and genes for studying specific facets of complex diseases.

Several approaches have been proposed to dissect diseases into molecular components. Given that disrupted biological processes are often shared among diseases [112,113,114], even those seemingly unrelated [115], it is promising to dissect diseases into dysregulated processes [116] or modules [117,118]. Additionally, phenotype ontologies, such as Human Phenotype Ontology (HPO) [119], are widely used tools for phenotype-driven disease analysis, enabling the breakdown of diseases into related phenotypes [120,121].

By leveraging dissected processes, modules, or phenotypes, we can identify combinations of research organisms that maximally capture the multifaceted nature of complex diseases. The methods discussed in this review, particularly in the section “How to identify analogous molecular components across species”, shed light on approaches for relating these dissected components to suitable research organisms. However, there remains a critical need for future approaches that can utilize analogous molecular components of complex diseases to guide the strategic selection of the most appropriate research organisms for specific disease aspects.

### From homology to agnology

Homology is widely used as the bridge to find functional similarities across species. However, the definition of homology does not include nor does it guarantee functional similarity. Several factors can lead to functional divergence between homologous genes. Examples include changes in non-coding regulatory sequences [15], reciprocal gene loss [122], and developmental system drift [123].

Moreover, homology restrains the power of predictive models to explore comprehensive functional relationships across divergent species. For instance, research organisms like the nematode *C. elegans* may not share a substantial number of gene orthologs with humans, resulting in a scarcity of genes available for model training and testing. This limited orthologous gene repertoire can hinder the ability of computational models to learn comprehensive functional relationships across species. Furthermore, orthologs alone can only explain a fraction of observed biological variations [97]. Therefore, it is crucial to incorporate species-/taxon-specific genes, i.e. agnologs, to construct more comprehensive models. Relying solely on homologous genes is limiting from the perspective of network biology, where species evolve through gain or loss of interactions, indicating that species-/taxon-specific genes can play a role in similar functional and regulatory programs [124].

While most methods discussed here suffer from the limitation of heavily relying on homology for knowledge transfer, approaches such as FKT [36], GenePlexusZoo [38] and SATURN [98] show how to include every gene in cross-species analyses, may they be homologous or not.

### Networks in more species and more contexts

Molecular networks are one of the most widely applied data types in cross-species knowledge transfer. Regardless of their representation (e.g., edgelists, adjacency matrices, or node embeddings), networks capture interactions among neighboring genes and provide mechanistic understanding to computational models. However, network-based methods also have limitations.

Most networks generated using experimental approaches are for humans or popular research organisms like mice. There is an urgent need to expand the repertoire of networks beyond these species to include non-traditional research organisms. Current methods, such as STRING [125] and those described in recent literature [126], use orthology information to infer “interologs”, i.e., conserved interactions between pairs of proteins that have interacting homologs in another organism [127,128]. For example, STRING utilizes high-confidence networks in humans and data-rich research organisms to derive protein-protein interaction (PPI) networks for over 1,000 species. However, the assumption that paired orthologous genes have conserved interactions across species may not always hold true due to interaction rewiring during the course of evolution. Additionally, limiting the scope to orthologous genes cannot infer interactions involving taxon-specific genes. Other previously introduced methods like FKT/IMP [36,129] use functional analogs to transfer knowledge across species, but these methods require experimental functional genomics data as a prior to generate networks, limiting their application to popular research organisms.

Another important future step in generating species-specific networks is the development of context-specific networks, particularly those with tissue or cell-type specificity. Context specificity plays a crucial role in biomedicine since disease-gene associations frequently arise from disrupted interactions among tissue-specific and cell lineage-specific processes under particular environmental conditions [51,130]. Unfortunately, the nuanced interactions that vary across tissues may not be fully captured by experimentally generated large-scale networks such as PPI. Coexpression networks have the potential to capture tissue specificity more effectively [131], but they tend to be noisy [132]. To obtain robust signals, current studies often extract only a small fraction of information for downstream analysis, such as the top 0.5% of co-expressed gene pairs [133]. This approach, however, results in the loss of significant information. Limited efforts have been made to build tissue-specific networks by integrating multi-modal functional genomic data [47,51] or to contextualize network representations by incorporating tissue-specific expression [134,135,136].

Recent advances in sequence-based deep learning models could help expand the repertoire of species-specific and context-specific networks. For example, AlphaFold-Multimer [137] can predict protein interactions based on sequences. ExPecto [138] and ExPectoSC [139] can predict tissue-/cell- specific regulatory landscapes based on DNA sequences alone. Thanks to the advancement of genome projects for non-model species, such as the Vertebrate Genomes Project [140], genome sequence resources are much richer than other genomics types such as transcriptomes or epigenomes. Leveraging the power of these sequence-based models and transfer learning techniques poses a promising way to expand networks beyond popular research organisms. However, we need to integrate phylogenetic information into transfer learning to consider the taxonomic-specific logic of gene regulation or protein interactions.

### Automated construction of ontologies and knowledge graphs

In this review, we have described the power of ontologies as a framework for transferring knowledge across species. Furthermore, combining various ontologies and annotations into knowledge graphs can provide new insights for translational biomedicine. The Monarch Knowledge Graph exemplifies this approach, integrating knowledge from 33 biomedical resources, including information from all major research organism databases [72]. Leveraging such knowledge graphs, we can employ advanced techniques like graph deep learning [141] to utilize information from research organisms and assist biomedical studies such as rare disease variant prioritization [142]. Integrating knowledge graphs with large language models can also help reduce “hallucinations” in AI-powered question-answering systems within the biomedical field [143].

Despite the power of ontologies and knowledge graphs in cross-species knowledge transfer, the generation of ontologies requires laborious curation by specialists. Manual curation may also introduce biases towards popular research areas. For instance, since zebrafish is a widely used research organism for developmental and embryogenic studies, GO terms annotated to zebrafish might be skewed towards these areas. Such differences in term annotation frequency could in turn bias downstream genomic analyses [144].

The capability of current large language models to effectively extract entities and relationships from the literature offers an efficient method for automatically generating ontologies and knowledge graphs. For example, SPIRE [145] can generate ontologies by processing text input and a user-provided schema that describes the desired structure of the ontology. While more effort is needed to maintain the quality and consistency of automatically generated ontologies, this approach holds great potential for significantly enriching ontologies across a wide spectrum of organisms, from well-studied to understudied research organisms and even non-model organisms.

### Better benchmarking studies for cross-species scRNAseq analyses

Mapping single-cell and single-nuclei transcriptomics (scRNA-seq/snRNA-seq) data across different species provides valuable insights into cell type evolution and facilitates cell type and cell state annotation [96,97]. However, most existing cross-species cell mapping algorithms are limited to closely related species and do not account for full homology [97], while considering all homologous genes are crucial when comparing species involving gene duplication at their common ancestors. For example, due to a genome duplication in the ancestor of teleost fishes, considering full homology is essential for cross-species integration of cell types between human and biomedical fish models such as zebrafish [146]. To overcome this limitation, algorithms such as SAMap [109] and SATURN [98] have been developed. While recent benchmarking studies highlight properties of these methods that are important for effective cross-species integration of scRNA-seq data [108], most of these methods were originally designed for batch corrections. Furthermore, since SAMap applied additional restrictions on within-species manifolds, SAMap was not systematically compared to other methods in current benchmarking studies. Thus, it is essential to conduct more comprehensive benchmarking of these methods to evaluate their performance on different types of scRNA-seq datasets and species with varying degrees of divergence and data curation. Additionally, the development of new methods for downstream analyses is crucial to extract meaningful biological insights from the growing mountain of single-cell/nuclei data.

### Extracting and curating knowledge from non-traditional research organisms

The majority of the organisms noted in this review are classic research organisms such as mouse, zebrafish, and the nematode worm, with non-traditional and emerging research organisms often being overlooked in biomedical studies. However, non-traditional research organisms have significant contributions to translational research due to their unique characteristics [147]. For instance, the axolotl (*Ambystoma mexicanum*), a neotenic salamander, shows remarkable regenerative abilities, making it a crucial research organism for regenerative medicine and developmental biology [148]. The spotted gar (*Lepisosteus oculatus*), a ray-finned fish with a slow evolutionary rate, has proven valuable in facilitating genomic comparison between teleost biomedical research organisms and the human genome [32]. The tunicate, *Ciona intestinalis*, is a close invertebrate relative of vertebrates. Its unique evolutionary position, simple body plan, and ease of manipulation make it highly suitable for studying embryonic development and morphogenesis [149]. However, challenges such as polyploidy, large genome sizes, genomic rearrangements, and taxon-specific cell types can make analyzing the genomic data from such species more difficult than classic research organisms. It is necessary to explore additional methods that can effectively transfer knowledge from and to non-traditional research organism data sets.

## Conclusions

In this review, we have discussed the current state of computational methods for cross-species knowledge transfer. We emphasize that these methods surpass simple comparisons of molecular profiles across species and highlight the utilization of orthogonal information sources such as phenotypic ontologies and molecular networks to facilitate cross-species knowledge transfer.

Our review provides valuable resources and insights into the advancements and challenges of methods for cross-species knowledge transfer among various research organisms and human. The resources summarized in this review will facilitate biomedical studies using research organisms, including traditional and non-traditional research organisms, by leveraging knowledge from human or extensively studied model systems.

There are still needs and plenty of room for further improvements and refinements of existing approaches. More advanced computational approaches are needed to identify a range of research organisms for studying different aspects of diseases. Instead of relying solely on homology as a bridge, expanding analyses to agnologs can enhance the effectiveness of cross-species knowledge transfer. To gain a more comprehensive understanding of diseases, we need networks from more species, and also need to incorporate context specificity, especially cell- and tissue-specificity, into networks, despite the inherent difficulties involved. To fully unlock the power of ontology-based knowledge transfer, we need methods to automatically generate high-quality and robust ontologies as well as knowledge graphs. As a rapidly growing field, it is essential to establish more benchmarks for state-of-the-art cross-species cell mapping between humans and evolutionary divergent research organisms. Currently, most methods are focused on the few well-studied research organisms, but greater attention and analytical methods should be employed to extract biomedical insights from non-traditional, emerging research organisms.

We propose that with the application of comprehensive computational approaches, the field will gain more exciting insights from big data of research organisms, ultimately enhancing our understanding of human biology and diseases.
